# Distinct Activities of Glycolytic Enzymes Identify Chronic Lymphocytic Leukemia Patients with a more Aggressive Course and Resistance to Chemo-Immunotherapy

**DOI:** 10.1016/j.ebiom.2018.05.030

**Published:** 2018-06-05

**Authors:** Georg Gdynia, Tadeusz Robak, Jürgen Kopitz, Anette Heller, Svetlana Grekova, Katarina Duglova, Gloria Laukemper, Monika Heinzel-Gutenbrunner, Cornelius Gutenbrunner, Wilfried Roth, Anthony D. Ho, Peter Schirmacher, Michael Schmitt, Peter Dreger, Leopold Sellner

**Affiliations:** a Institute of Pathology, University Hospital Heidelberg, Heidelberg, Germany; b Medical University of Lodz, Copernicus Memorial Hospital, Lodz, Poland; c Department of Child and Adolescent Psychiatry, University Hospital Marburg, Marburg, Germany; d MH Statistical Consulting, Marburg, Germany; e Institute of Pathology, University Medical Center of the Johannes Gutenberg University Mainz, 55131 Mainz, Germany; f Department of Medicine V, University Hospital Heidelberg, Heidelberg, Germany; g National Center for Tumor Diseases (NCT), German Cancer Consortium (DKTK), Heidelberg, Germany

**Keywords:** CLL, Metabolism, PK M2, LDH, High-risk

## Abstract

A higher capacity to grow under hypoxic conditions can lead to a more aggressive behavior of tumor cells. Determining tumor activity under hypoxia may identify chronic lymphocytic leukemia (CLL) with aggressive clinical course and predict response to chemo-immunotherapy (CIT). A metabolic score was generated by determining pyruvate kinase and lactate dehydrogenase, key enzymes of glycolysis, ex vivo in primary CLL samples under normoxic and hypoxic conditions. This score was further correlated with clinical endpoints and response to CIT in 96 CLL patients. 45 patients were classified as metabolic high risk (HR), 51 as low risk (LR). Treatment-free survival (TFS) was significantly shorter in HR patients (median 394 vs 723 days, *p* = .021). 15 HR patients and 14 LR patients received CIT after sample acquisition. HR patients had a significantly shorter progression-free survival after treatment compared to LR patients (median 216 days vs not reached, *p* = .008). Multivariate analysis evaluating age, IGHV, *TP53* deletion or mutation and 11q22–23 deletion besides the capacity of tumor cells to grow under severe hypoxic conditions identified the metabolic profile as the strongest independent risk factor for shorter TFS (hazard ratio 2.37, *p* = .011). The metabolic risk can provide prognostic and predictive information complementary to genetic biomarkers and identify patients who might benefit from alternative treatment approaches.

## Highlights

• The activity of distinct glycolytic enzymes can identify CLL patients with resistance to chemo-immunotherapy
• The activity of distinct glycolytic enzymes can identify CLL patients who may benefit from specific pathway inhibitors
• We provide a tool for the evaluation of specific glycolytic enzymes in primary CLL cells for clinical diagnostics

## 1 Introduction

Different malignancies contain subpopulations of tumor cells that can grow in hypoxic (severely oxygen deficient) microenvironment [1,2]. These cancer cells can be responsible for poor outcome and resistance to radiation and chemotherapy [3]. Cancer cells growing under hypoxia produce energy and building blocks for macromolecules by anaerobic glycolysis, independent from mitochondrial (oxygen dependent) respiration [4]. Increased activities of the pyruvate kinase (PK) isoform M2 and of lactate dehydrogenase (LDH) allow faster incorporation of glucose metabolites into biomass facilitating cancer cell growth without oxygen [5,6]. Previously we have shown that oxygen-independent growth renders cancer cells resistant to Natural Killer (NK) cells. Cancer withstanding metabolic killing by the innate immune system use both, PK (M2) and LDH, to up-regulate anabolism and energy supply via glycolysis [7,8]. In summary, the capacity of cancer cells to grow fast under hypoxia correlates with resistance to therapy and elimination by immune cells. However, the ability of glycolysis in tumor cells is still unexploited for stratification and treatment of cancer patients. Here we provide a tool that could be easily implemented in clinical diagnostics. Employment of LDH and PK M2 under hypoxia (compared to normoxia) indicates fast hypoxic (anaerobic) cellular growth [5,9]. We developed an assay mimicking the hypoxic cancer microenvironment ex vivo by cultivation of primary chronic lymphocytic leukemia (CLL) cells from peripheral blood under hypoxia with PK M2 and LDH activity as readout.

Intensive chemo-immunotherapy (CIT) with fludarabine, cyclophosphamide and rituximab (FCR) [10] or bendamustine and rituximab (BR) [11] has dramatically improved the outcome of CLL with potential for long-term remissions. However, substantial variability can be observed in the course of CLL. Some patients are asymptomatic at time of diagnosis and do not need treatment for a long period. On the other hand, subgroups of patients develop rapid disease progression and early therapy resistance. A major issue in CLL is the identification of these patients who do not benefit from intensive CIT. So far, only *TP53* disruption (del17p13 and *TP53* mutation) is an established predictive marker for CIT refractoriness. These patients rather benefit from novel treatment approaches in CLL such as inhibitors of the B-cell receptor pathway (BCRi), e.g. the BTK inhibitor ibrutinib [12] and the PI3Kδ inhibitor idelalisib [13], or antiapoptotic proteins, e.g. the Bcl-2 inhibitor venetoclax [14]. However, a large proportion of CIT refractory patients do not harbor a disruption in *TP53*. Despite intense efforts, no reliable markers are available to predict response towards CIT in *TP53* wild-type patients [15]. The aim of the current study was to assess feasibility as well as prognostic and predictive value of PK M2 and LDH activity after cultivation of leukemia cells under hypoxia for the identification of CLL patients with aggressive clinical courses and resistance to CIT.

## 2 Patients and methods

### 2.1 Sample extraction and clinicopathologic data

The study sample consisted of consecutive 96 patients diagnosed with CLL who presented at the University Hospital Heidelberg between 2013 and 2014. Peripheral blood mononuclear cells (PBMCs) were isolated by a Ficoll gradient. The research was approved by the Ethics Committee of the University of Heidelberg (S-356/2013 and S-254/2016). Informed consent was obtained from all patients in accordance with the Declaration of Helsinki.

### 2.2 Genetic aberrations

Chromosomal aberrations by fluorescence in situ hybridization (FISH) were obtained from medical reports and were available for del [11](q22.3) (*n* = 92), trisomy 12 (*n* = 91), del [13](q14) (n = 92) and del [17](p13) (n = 91). Targeted sequencing for genetic aberrations in *NOTCH1*, *SF3B1,* and *TP53* was performed on a GS Junior benchtop sequencer (Roche, Penzberg, Germany) as described before [16].

### 2.3 Cell lines

The CLL cell line Mec-1 was obtained from the DSMZ (German Collection of Microorganisms and Cell Cultures, Braunschweig, Germany; RRID: CVCL_1870) and cultured in RPMI 1640 (Thermo Fisher Scientific, Waltham, MA, USA) supplemented with 2 mM l-glutamine (Thermo Fisher Scientific) and 10% heat-inactivated fetal bovine serum (FBS) (Thermo Fisher Scientific) at 37 °C.

### 2.4 Cytotoxicity assay

Cytotoxicity measurements were performed under very low oxygen conditions in 96-well plates using the ATP-based CellTiter Glo assay (Promega, Madison, WI, USA). Cells were cultured for 24 h with or without fludarabine (Sigma-Aldrich, St. Louis, MI, USA). In addition, PK M2 activity was modulated by PM2-tide (GGAVDDDpYAQFANGG; Enzo Life Sciences, Farmingdale, NY, USA; 10 μM) or DASA (1-(2,6-Difluorophenylsulfonyl)-4-(2,3-dihydrobenzo[b][1,4]dioxin-6-ylsulfonyl)piperazine; Merck Millipore, Burlington, MA, USA; 10 μM). The number of viable cells was calculated as % of the untreated control.

### 2.5 Glucose flux and lactate efflux

Glycolysis was measured by monitoring the conversion of 5- ^3^H-Glucose to ^3^H_2_O as described by Liang et al. [17]. In brief, cells were washed in PBS and resuspended in 1 ml Krebs buffer containing 10 mM glucose, and spiked with 370 MBq 5-^3^H-Glucose (Hartmann Analytic, Braunschweig, Germany). Following incubation for 1 h at 37 °C diffusion through a PCR vial was used to separate ^3^H_2_O formed by glycolysis. Radioactivity was determined in a liquid scintillation counter (TRICARB 2900, PerkinElmer, Waltham, USA). Lactate efflux was quantified by spectrophotometric assay as described by Brandt et al. [18].

### 2.6 Quantitative reverse transcriptase polymerase chain reaction (qRT-PCR)

qRT-PCR analysis was performed with either 1:5 or 1:10 diluted cDNA and analyzed in triplicates using the StepOne Plus thermo cycler (Applied Biosystems, Foster City, CA, USA). The cycling program was performed as follows: 95 °C for 10 min, followed by 40 cycles at 95 °C for 15 s and 60 °C for 1 min. Gene expression was normalized to two variants of the housekeeping gene 18S rRNA and data were quantified by StepOne Software v2.1. Fold change of expression was determined by the ΔΔCt method as described by Schmittgen and Livak et al. [19] The primer pairs used are listed in the supplementary methods.

### 2.7 Phosphofructokinase and hexokinase activity

Phosphofructokinase and hexokinase activity were assayed as described in Teslaa et al. [20] using homogenates from 10 [6] Mec-1 cells.

### 2.8 Immunoblot analysis and protein preparation

Immunoblotting was performed according to standard procedures by SDS–polyacrylamide gel electrophoresis. Cells were lysed in lysis buffer P (20 mM Tris-HCl (pH 7.4), 137 mM NaCl, 10% (*v*/v) glycerine, 1% Triton X-100, 2 μM EDTA, 100 mM phenylmethylsulfonyl fluoride and protease inhibitors (Complete mini from Roche). Lysates were centrifuged at 14,000 *g* (10 min) at 4 °C. Total protein was measured by the Bradford (Bio-Rad, Hercules, CA, USA) method. Soluble protein was resolved by SDS–polyacrylamide gel electrophoresis, blotted onto nitrocellulose and incubated with one of the following antibodies: rabbit polyclonal anti PKM2 (1:1000, Cell Signaling, Danvers, MA, USA; 4053S; RRID: AB_1904096), rabbit polyclonal anti LDHA (1:1000, Cell Signaling; 2012S; RRID: AB_2137173), rabbit polyclonal anti GAPDH (1:1000, Santa Cruz Biotechnology, Dallas, TX, USA; sc-365,062; RRID: AB_10847862) and HIF1 alpha (1:1000, StressMarq Biosciences, Victoria, Canada; SMC-184; RRID: AB_2570396). Appropriate secondary antibodies (1:3000, horse-radish peroxidase-conjugated, #170–6515 (goat anti rabbit IgG; RRID: AB_11125142) and #170–6516 (goat anti mouse IgG; RRID: AB_11125547)) were from Bio-Rad. Visualization was done by enhanced chemiluminescence technique (GE-Healthcare, Little Chalfont, UK). Uncropped versions of the membranes are shown in Supplementary Fig. S1/2.

### 2.9 Metabolic score

#### 2.9.1 Preanalytic

Two 3 cm petri dishes per patient were filled with 3 ml RPMI 1640 (Life Technologies, Paisely, UK) and 1*10^7^ cells. One was wrapped with an oxygen impermeable shell (GasPakᵀᴹ EZ, Becton Dickinson, New Jersey, USA) to generate anaerobic conditions. Both dishes were incubated overnight (16-24 h) at 37 °C and 5% CO_2_ (hypoxic (anaerobic) (Hx) and normoxic (Nx) sample). There was no significant difference in the total number of viable CLL cells between hypoxic and normoxic conditions after overnight culture. After incubation, cells were washed with PBS and resolved in 500 μl buffer solution (50 mmol/l KCl, 5 mmol/l MgCl_2_, Tris 20 mmol/l, 250 mmol/l sucrose in ddH_2_O, pH 7.4). To extract cytosolic proteins, the cell suspension was homogenized by ultrasonication (Diagenode Bioruptor® Sonication System, Diagenode, Seraing, Belgium). The enzyme activity of the three enzymes PK-la, PK-ha, and LDH was analyzed in the supernatant.

#### 2.9.2 Analytic

After determination of protein concentrations with Bradford Reagent (Bio Rad, Munich, Germany) the supernatants were adjusted to 0.1 μg/μl with homogenization buffer. Three aliquots (10 μl each) of the dilutions were transferred to 96-well plates (Greiner, Frickenhausen, Germany). PK-la and PK-ha activities were measured in a coupled enzyme assay with LDH, while for LDH activity decrease of NADH was monitored directly as described before [8]. All components of the assay were included in 190 μl of starting solution consisting of 10 mmol/l Phospho(enol)pyruvic acid (PEP), 1 mmol/l Adenosine-5′-diphosphate (ADP), 0.5 mmol/l NADH, 50 mmol/l KCl, 5 mmol/l MgCl_2_, 20 mmol/l Tris 12.5 mmol/l Sucrose, and 2 Units LDH (all Sigma Aldrich/Merck) for PK-la. The start reagent of PK-ha was identical to the start reagent of PK-la, except that the concentration of PEP was reduced to 0.1 mmol/l. For measurement of LDH activity the start solution was composed of 1 mmol/l Pyruvate (Sigma Aldrich/Merck), 0·5 mmol/l NADH, 50 mmol/l KCl, 5 mmol/l MgCl_2_, 20 mmol/l Tris, and 12.5 mmol/l Sucrose. All start reagents were adjusted to pH 7.4. After adding the starting reagent, oxidation of NADH was monitored at 340 nm for 30 min at 37 °C in a microplate reader (VICTOR × 2030, Perkin Elmer). With given Km values of 2.1 mM (PK la), 0.05 mM (PK ha) and 0.12 mM (LDH) [21] and using minimum 2fold substrate concentrations (PEP, Pyruvate) enzymes in the assay were always saturated and thus measured within the linear range with no substantial change of substrate concentration during the assay time. In addition, positive and negative controls were run in separate wells of the plate. 5 mU/10 μl PK M2 Pyruvate Kinase Type VII (Sigma Aldrich/Merck) were applied as positive controls for PK-la and PK-ha, 3 mU/10 μl as positive control for LDH. 10 μl of homogenization buffer served as negative control. All samples were measured in duplicates.

### 2.10 Metabolic score calculation

The metabolic score (MS) is a sum of (a) the anaerobic:aerobic capacity ratio (inverse value) and (b) the tumor PK M2:PK ratio. Increased (a) facilitates cellular growth/energy homeostasis under oxygen deficiency, increased (b) accelerates growth in fast proliferating and/or cancer cells, in particular under oxygen deficiency [9]. The change in enzyme activities upon severe hypoxic stimulation indicates increased glycolytic flux under hypoxia.

(a) $$\text{Anaerobic}:\text{aerobic capacity}=\frac{\mathrm{LDH}}{\mathrm{PK}\;\text{high}\;}$$

(b) $$\text{Tumor}\;\mathrm{PK}\;M2:\mathrm{PK}\;\text{ratio}=\frac{\mathrm{PK}\;\mathrm{low}\;\text{affinity}}{\mathrm{PK}\;\text{high affinity}}$$

(c) $$\mathrm{Sum}\;\text{of}\;\left(a\right)+\left(b\right)=\frac{\mathrm{PK}\;\mathrm{low}\;\text{affinity}+\mathrm{LDH}}{\mathrm{PK}\;\text{high affinity}}$$

Enzyme activity was measured at 0 min and 30 min after beginning of the reaction. Duplicates were averaged and inserted into the MS equation. Blank corrected endpoint data utilized to generate the metabolic scores for all patients are provided in a supplementary excel file. No change in the ratio of the ‘anaerobic’ (numerator) versus ‘aerobic’ (denominator) enzyme activity upon hypoxic cultivation results in a value of $2.0=\frac{1+1}{1}$. The change in activity upon very low oxygen cultivation results in a higher (increased PK la and/or LDH) or lower (increased PK ha) MS than 2.0. A significant deviation outside the range 2.0 ± 0.3 (0.3~3xSD) indicates better cellular growth under hypoxia. Increased activity of the PK M2 and increased activity of LDH allows faster incorporation of glucose metabolites into biomass [6,9]. PK M2 exists as a dimer (so called tumor PK M2) with low affinity (PK-la) and as a tetramer with high affinity (PK-ha) to its substrate PEP. The tetrameric PK M2 is characterized by a high Km for PEP. Dimeric PK M2 is virtually inactive at physiological PEP levels allowing differentiation of both forms by using very high (10 mM) and low (100 μM) amounts of PEP in the enzymatic assay [8]. Increased activity of overall PK M2 enzyme results in increased glucose flux and cancer cell growth, whereby a higher abundance of PK low affinity over PK high affinity is even more advantageous for rapid macromolecule synthesis [9]. Validation of the test procedure is described in the supplementary methods.

### 2.11 Statistical analyses

Statistical analyses were performed using statistical software SAS(r) 9.4 (SAS Institute, Cary, NC, USA), R 3.2.0 (http://www.R-project.org) and Excel (Microsoft, Redmond, WA, USA).

Statistical significance in the preclinical cell line model was calculated with a two-sided Student's *t*-test. Error bars are indicating standard deviations (SD).

The simultaneous influence of several factors including the MS on the defined endpoints was analyzed using Cox regression, Kaplan-Meier curves and the Logrank-Test. Endpoints were defined according to iwCLL criteria [22].

The MS was used in a dichotomized (D) version: D-MS = 0/1 if |MS-2| ≤ / > 0.3 (D-MS = 0 is equivalent to 1.7 ≤ MS ≤ 2.3). Only for ROC-analysis a variable cut off *c* for |MS-2| was used instead of the fixed cut off value 0.3.

Patient characteristics in the two groups defined by the D-MS were compared by means of Fisher's Exact test for categorical parameters and *t*-tests for metric parameters.

To define the diagnostic sensitivity and specificity of the MS CLL test, the following equations were used: Sensitivity [%] = 100 x (number of high risk patients (defined as progress within 2 years after CIT) with |MS-2| > 0.3) / total number of high risk patients. Specificity [%] = 100 x (number of low risk patients (defined as no progress event (within 2 years) after CIT) with |MS-2| > 0.3) / total number of low risk patients.

## 3 Results

### 3.1 Distinct activities of glycolytic enzymes are defining sensitivity towards chemotherapy

**Fig. 1:**
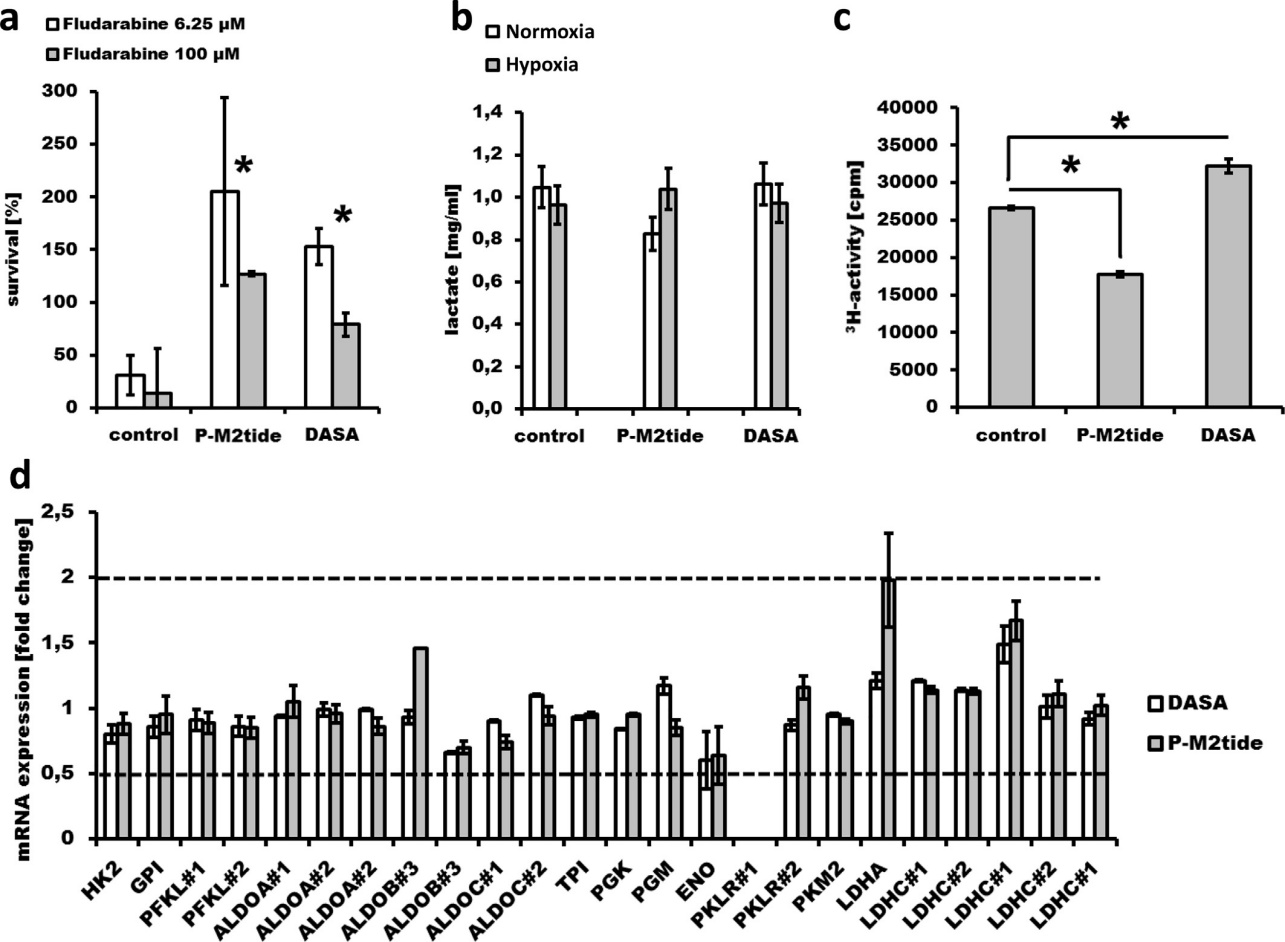
Glycolytic enzymes and glucose flux in Mec-1 cells. 10 [4] Mec-1 cells were treated with 10 μM P-M2tide or 10 μM DASA, respectively (both with no cytotoxicity towards Mec-1 cells), and with fludarabine (simultaneously, under hypoxia for 24 h, *n* = 4; A). Viability was defined as % luminescence of the untreated control. Lactate efflux after 24 h was measured using 10^6^ Mec-1 cells (*n* = 2; B). 5-^3^H-glucose turn-over was assessed after treatment with PM2-tide or DASA (under hypoxia, 24 h, *n* = 3; C). For qRT-PCR fold change of expression (compared to untreated control) of glycolytic Iso−/enzymes Hexokinase (HK), Glucose-6-phhosphate isomerase (GPI), Phosphofructokinase (PFKL, two different primers), Aldolase A/B/C (ALDOA, ALDOB and ALDOC each two different primers), Triose-phosphate isomerase (TPI), Phosphoglycerate mutase (PGM), Enolase (ENO), Pyruvate kinase (PKLR two different primers (PKLR was not detectable with primer #1); PKM2) and Lactate dehydrogenase (LDHA, LDHC with two primers (#1 and #2) and repetitive testing) was measured in untreated cells and after treatment with P-M2tide or DASA (under hypoxia, 24 h, *n* = 3; D). A minimum of 2-fold change (relative quantification of more than two or <0.5; dashed lines) was considered significant. Error bars are indicating standard deviation. Statistical significance was calculated with a two-way *t*-test. Significance is represented as * for *p*-values <.05.

According to our hypothesis that distinct activities of PK M2 and LDH in leukemia cells during cultivation in severe hypoxic conditions may define sensitivity or resistance towards chemotherapy we generated preclinical mechanistic evidence with the well-characterized CLL model cell line Mec-1. Mec-1 cells display *TP53* mutations [23], an established predictive marker for chemo- and/or immunotherapy resistance in CLL. Under hypoxia, Mec-1 cells are still sensitive to fludarabine, especially in very high concentrations (100 μM). However, after pharmacological modification of PK M2 activity, Mec-1 cells develop resistance towards fludarabine under hypoxia (*p* = .009/*p* = .0000004 (for 6.25 μM or 100 μM fludarabine), control vs. PM2-tide and *p* = .00008/*p* = .001 (for 6.25 μM or 100 μM fludarabine), control vs DASA; Fig. 1A). Modulation of PK M2 activity was performed in two directions under hypoxia: (i) pharmacological inhibition of PK M2 by specifically blocking the PK M2 tetramer by P-M2tide and (ii) pharmacological activation of PK M2 by the specific activator DASA, and led to significant change of the metabolic score described in this work (Supplementary table 1).

In addition, lactate levels in the media of Mec-1 cells were measured under normoxic and hypoxic conditions, and both with and without pharmacological modulation of PK M2 activity (Fig. 1B). There was no significant change in lactate levels (from normoxia to hypoxia) after modulation of PK M2 activity compared to untreated controls. Thus, this finding may be interpreted that the glucose consumption did not change. However, further analysis of the glucose flux (at the Enolase reaction step) revealed 33% decline of glucose flux after pharmacological inhibition of PK M2 by P-M2tide and 21% increase of glucose flux after pharmacological activation of PK M2 by DASA (*p* = .00004, control vs PM2-tide and *p* = .0005 control vs DASA; Fig. 1C). Taking into account that glucose converted to lactate was unchanged, these results demonstrate that by modification of PK M2 activity, a major portion of intracellular glucose in Mec-1 cells is differently apportioned for ancillary biosynthetic reactions (e.g. pentose phosphate, hexosamine biosynthetic, glycerolipid, alanine and oxaloacetate synthesis pathways) [24] above and down-stream of Enolase under hypoxic conditions. This strongly suggests that after modification of PK M2 activity Mec-1 cells operate with an increased glucose carbon pool amenable to entry in anabolic pathways under hypoxic conditions.

To exclude that the abundance of other glycolytic enzymes are involved in the PK M2-dependent phenotype, mRNA abundance of different glycolytic enzymes were assessed in Mec-1 cells after pharmacological modulation of PK M2 (Fig. 1D). There were no significant differences of mRNA levels of glycolytic enzymes in Mec-1 cells after PK M2 modulation. These results support the hypothesis that post-translational modification of the glycolytic enzyme PK M2 (e.g. by pharmacological modulation with P-M2tide or DASA), without any detectable change in mRNA levels of PK M2 and of any of the other glycolytic enzymes, may be sufficient to make leukemia cells resistant to chemotherapeutic agents such as fludarabine.

**Fig. 2:**
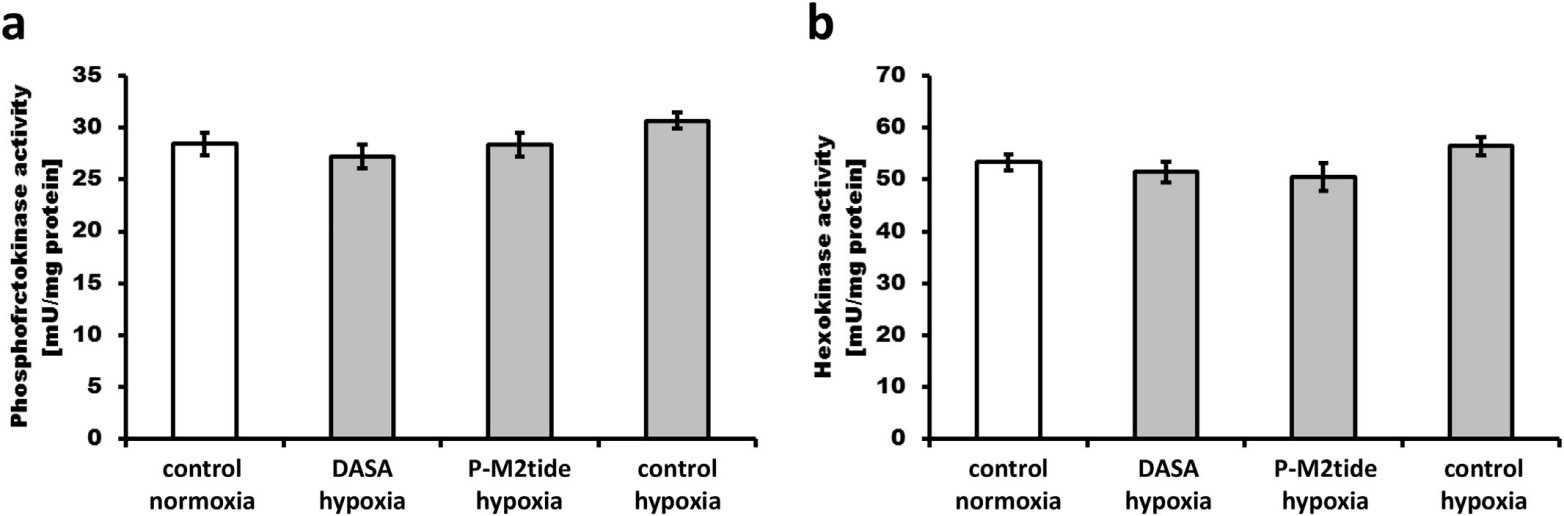
Activity of phosphofructokinase and hexokinase in normoxia and hypoxia after pharmacological modulation of PK M2. Homogenates from 10^6^ Mec-1 cells used for the experiments shown in Fig. 1B and Fig. 1D were analyzed for specific enzyme activities (A, B). There were no significant changes in activities.

To assess if the activity of other enzymes may be responsible for the leukemia cell phenotype under hypoxic conditions, enzyme activities of two other rate-limiting glycolytic enzymes, hexokinase and phosphofructokinase, were assessed in normoxia and hypoxia after pharmacological modulation of PK M2 (Fig. 2). No significant differences in activity of hexokinase and phosphofructokinase could be observed between normoxia and hypoxia as well as with or without DASA or P-M2tide.

We also wondered if the specific, direct pharmacological modification of PK M2 activity resulting in resistance to fludarabine under hypoxia was accompanied by changed levels of HIF1-alpha. HIF1-alpha, that often activates the switch-on of glycolysis under hypoxia, was evenly degraded under hypoxia (compared to normoxia) in control, PM2-tide and DASA treated Mec-1 cells (Supplementary Fig. S1). Although there were no apparent significant changes between control and treated cells, HIF1-alpha degradation was rather unexpected, but it is known that prolonged hypoxia (12*h*) can result in strong degradation of HIF1-alpha [25].

In summary, pharmacological modulation of PK M2 activity, resulting in a significant change of the metabolic score, is sufficient to render Mec-1 cells resistant to fludarabine under severe hypoxia. In addition, a genetic high risk status (del [17](p13) /*TP53* mutation) does not per se protect leukemia cells from fludarabine-mediated cytotoxicity under severe hypoxia. These preclinical data lead us to further evaluate the impact of distinct activities of glycolytic enzymes on the clinical outcome of CLL patients.

### 3.2 Characteristics of the CLL test cohort

96 patients were prospectively enrolled in our trial. 45 patients were classified as MS HR, 51 as LR according to the occurrence of a substantial subpopulation of hypoxic cells. MS of each individual patient with the classification into HR and LR are listed in Supplementary Table 2. Clinical characteristics are shown in Table 1. Both metabolic risk groups showed similar clinical parameters including cytogenetic abnormalities, *TP53* mutation, *IGHV* status, lymphocyte doubling time as well as previous treatments. HR patients had a trend towards a higher WBC at sample acquisition (Table 1). Consistent with the fact that both risk groups had similar clinical parameters there was no significant association (0.091 > *p* < 0,730) between absolute MS deviation and del(17p) and/or *TP53* mutation, *IGHV* status, 11q22–23 (del(11q)), treatment before sample extraction, WBC, lymphocyte doubling time or PB lymphocytes (Supplementary Fig. S3). Of note, although PK M2 and LDH enzyme activities in individual patients were changed (normoxia compared to hypoxia), the protein expression of these and other glycolytic enzymes was unchanged (normoxia compared to hypoxia) as shown in western blot analysis of PK M2, LDH and GAPDH in 4 CLL study patient samples classified as MS HR and 4 classified as MS LR (Supplementary Fig. S2).

**Table 1:** Patient characteristics.

	**D-MS HR (n** = 45)	D-MS LR (n = 51)	p-value
MS, median (range)	1.62 (0.78–3.45)	1.95 (1.7–2.28)	0.382
Age at diagnosis, median (range) [years]	58 (31–82)	64 (38–83)	0.141
Age at sample, median (range) [years]	66 (38–83)	68 (42–88)	0.220
Age ≥ 65 years, n (%)	26 (58)	30 (59)	0.473
Age ≥ 75 years, n (%)	6 (13)	13 (25)	0.123
Sex, male/female, n	29/16	28/23	0.229
Median time from diagnosis to sample, median (range) [months]	72 (0–194)	56 (0–247)	0.580
WBC at sample, median (range) [/nl]	70 (19–262)	55 (17–234)	0.069
PB lymphocytes, median (range) [%]	93 (74–100)	91 (67–100)	0.121
Previous treatment, n (%)	13 (29)	11 (22)	0.203
Prior treatments, median (range)	0 (0–9)	0 (0–4)	0.299
Treatment after sample, n (%)	22 (49)	20 (39)	0.227
Novel treatment after sample, n (%)	7 (16)	8 (16)	0.610
Ibrutinib, n (%)	6 (13)	4 (8)	0.293
Idelalisib, n (%)	3 (6)	4 (8)	0.570
ABT-199, n (%)	0	1 (2)	0.531
Binet at diagnosis, A/B/C/unknown [n]	26/11/1/7	38/5/2/6	0.193
LDT at sample, median (mean; range) [months]	12 (20; 1–62)	26 (34; 3–80)	0.166
Cytogenetic abnormalities			
del11q22–23, n (%)	8/45 (18)	6/47 (13)	0.570
Trisomy 12, n (%)	6/44 (14)	11/47 (23)	0.287
del13q14, n (%)	31/44 (70)	28/48 (58)	0.321
del17p13, n (%)	7/44 (16)	4/47 (9)	0.259
*IGHV* unmutated	18/35 (51)	16/38 (42)	0.681
*NOTCH1* mutation, n (%)	4/41 (10)	3/44 (7)	0.460
*SF3B1* mutation, n (%)	6/40 (15)	5/44 (11)	0.755
*TP53* mutation, n (%)	9/41 (22)	5/45 (11)	0.356
Del17p13 and/or *TP53* mutation	10/40 (25)	8/42 (19)	0.795

### 3.3 Correlation of the metabolic score with the clinical course

**Fig. 3:**
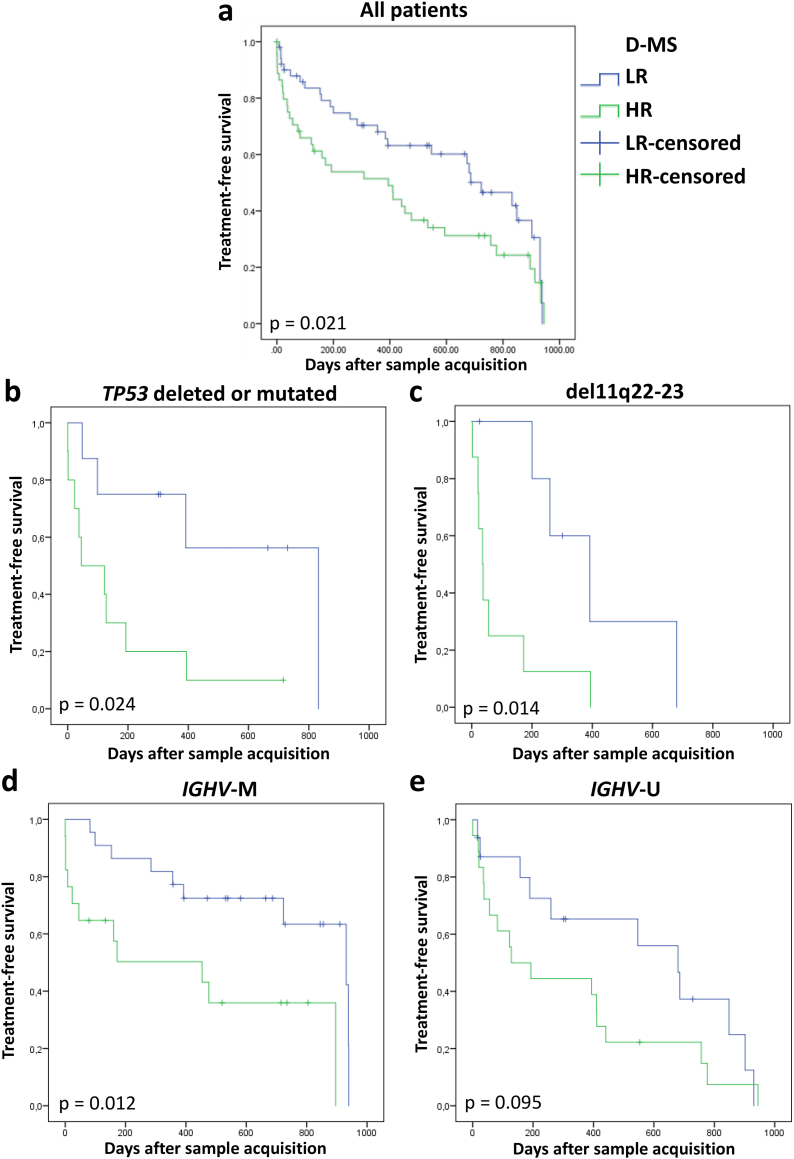
Treatment-free survival according to dichotomized metabolic score (D-MS). Analysis of all CLL patients (HR *n* = 45, LR *n* = 51; A), as well as subgroups that are *TP53* deleted or mutated (HR *n* = 8, LR *n* = 10; B), with del11q22–23 (HR n = 8, LR *n* = 6; C), *IGHV*-M (HR *n* = 17, LR *n* = 22; D) and *IGHV*-U (HR *n* = 18, LR *n* = 16; E).

Treatment-free survival (TFS) measured from the day of sample acquisition to the first day of a CLL-specific treatment was significantly shorter in HR patients (median TFS 394 vs 723 days, *p* = .021; Fig. 3A). Subgroup analysis revealed significant adverse effects of metabolic HR even in patients harboring high risk genetic aberrations including *TP53* deletion or mutation (median TFS HR 45 vs LR 832 days, *p* = .024, Fig. 3B) as well as del(11q) (median TFS HR 36 vs LR 392 days, *p* = .014; Fig. 3C). Metabolic HR was associated with reduced TFS in both *IGHV* mutated (*IGHV*-M; HR 453 vs LR 931 days, *p* = .012; Fig. 13D) and *IGHV* unmutated (*IGHV*-U; HR 128 vs LR 680 days, *p* = .095; Fig. 3E) patients. Analysis of overall survival (OS) showed a trend towards shorter OS in the HR patient cohort (HR vs LR days, *p* = .107; Supplementary Fig. S4).

### 3.4 Impact of the metabolic score on treatment response

**Fig. 4:**
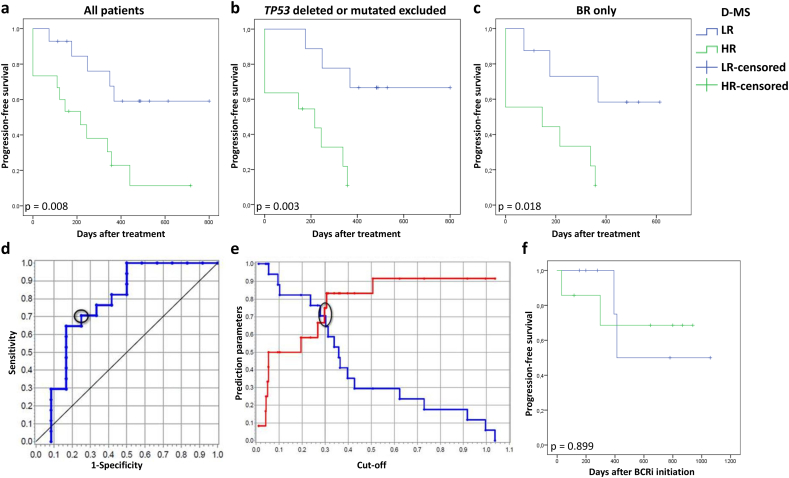
Progression-free survival after treatment with chemo-immunotherapy according to dichotomized metabolic score (D-MS). Analysis of all CLL patients treated with chemo-immunotherapy (HR *n* = 15, LR *n* = 14; **A**). Subgroup analysis after exclusion of patients that are *TP53* deleted or mutated (HR *n* = 11, LR *n* = 9, **B**). Subgroup analysis of patients treated with the combination of bendamustine with rituximab (BR; HR n = 9, LR n = 8; C). Based on the results of CLL patient sample analysis, the cut-off of the test value |MS-2| was variated in the sense of an ROC analysis (HR n = 15, LR n = 14). It was ascertained that the sensitivity and the specificity were similar at a value of 0.3 correlating about 75% (D). The discriminatory performance of the test value |MS-2| is shown in the ROC curve with an area under curve of 0.77 (HR n = 15, LR n = 14; E). PFS was determined in CLL patients who were treated with BCRi after sampling (n = 14, ibrutinib/idelalisib HR n = 5/2, LR n = 3/4; F).

15 HR patients and 14 LR patients received CIT after sample acquisition. CIT included bendamustine in combination with rituximab (BR), cyclophosphamide / doxorubicine / vincristine / prednisolone in combination with rituximab (R-CHOP), chlorambucil in combination with rituximab (R-CBL) or obinutuzumab (G-CBL) and fludarabine / cyclophosphamide in combination with ofatumumab (O-FC). Progression-free survival (PFS) measured from start of first treatment after sample acquisition was significantly shorter in HR patients compared to LR patients (median PFS HR 216 days vs LR not reached, *p* = .008; Fig. 4A). It is known that patients with *TP53* aberrations respond poorly to CIT. Additional analysis excluding patients with *TP53* deletion or mutation confirmed significantly shorter PFS in metabolic HR patients (median PFS HR 216 days vs LR not reached, *p* = .003; Fig. 4B). Out of the CLL patients receiving CIT, nine HR and eight LR patients received BR. HR CLL receiving BR had a significantly shorter PFS compared with LR patients (median PFS HR 146 days vs LR not reached, *p* = .018; Fig. 4C). Diagnostic sensitivity and specificity of the MS for identification of high-risk CLL (progress within 2 years after CIT) were well-balanced: sensitivity = 71%; specificity = 75% (Fig. 4D). Compared to single enzyme activities (PK la/ha, LDH) MS had the highest AUC (c-statistics 0.77, *p* = .015) and thus was best at prediction of high-risk CLL (Supplementary Table 3). In order to evaluate if the outcome of metabolic HR patients was also worse if they were treated with specific pathway inhibitors, we determined PFS in patients who received BCRi. In this preliminary analysis, there were no differences between the two metabolic risk groups after treatment with BCRi (LR median 736 vs HR not reached, *p* = .899).

### 3.5 Multivariate analysis for treatment free survival

Multivariate analysis evaluating age, *IGHV* gene mutation status, *TP53* deletion or mutation and del(11q) besides the capacity of tumor cells to grow under hypoxic conditions was performed for TFS (Table 2). D-MS was identified as the most significant independent risk factor for shorter TFS (hazard ratio 2.37, *p* = .011). Besides D-MS, del(11q) was also accompanied by shorter TFS (hazard ratio 2.52, *p* = .03). *IGHV* status and *TP53* disruption did not significantly correlate with TFS in our cohort. This can be explained by the relatively short observation time where more subtle effects on TFS may not have turned out significant.

**Table 2:** Multivariate analysis for treatment-free survival (TFS).

Variable	Hazard ratio	95% CI	p-value
Age at sample acquisition	1.00	0.97–1.03	0.876
D-MS HR (<1.7 or > 2.3)	2.37	1.22–4.63	0.011
IGHV-U	1.48	0.73–3.01	0.277
del17p13 and/or *TP53* mutated	1.50	0.69–3.27	0.306
del11q22–23	2.53	1.09–5.87	0.030

Abbreviations: CI = Confidence Interval

## 4 Discussion

Despite novel treatment options with specific pathway inhibitors, CIT is standard of care in CLL. The identification of CLL patients who may not benefit from CIT is a major clinical challenge. Besides alteration in *TP53* there are currently no reliable markers available that can predict CIT resistance. In our pivotal trial we studied the hypoxic phenotype of CLL cells and its prognostic and predictive value.

In a preclinical lymphoid malignancy model with Mec-1 cells we provide evidence that PK M2 and LDH activity are crucial for sensitivity or resistance towards chemotherapeutic agents such as fludarabine. These findings lead us to evaluate the impact of PK M2 and LDH activity in primary CLL cells under hypoxic conditions on the clinical outcome of CLL patients in a translational approach.

Cancer cells can escape elimination by immune cells and chemotherapeutic agents by residing in hypoxic niches. We and others have shown that immune cells operate less efficiently in an oxygen-depleted microenvironment and preferably eliminate oxygen-dependent cancer cells requiring intact mitochondrial DNA (mtDNA) and a functioning respiratory chain [8,26]. mtDNA is more susceptible to damage by chemotherapeutic agents than nuclear DNA. Patients clinically refractory to bendamustine or fludarabine have higher inactivating mutations of mtDNA [27]. Furthermore, efficient mitochondrial respiration is not possible without functional p53 that is crucial for assembly of the mitochondrial complex IV of the respiratory chain (cytochrome *c* oxidase) by promoting expression of the gene encoding SCO2 (synthesis of cytochrome *c* oxidase protein) [28]. *TP53* is often mutated or deleted in refractory CLL. CLL cells circulating in normoxic conditions can be selectively killed by inhibition of the mitochondrial respiratory chain and induction of ROS overproduction [29]. The monoclonal CD20 antibody rituximab sensitizes leukemic cells to anticancer drugs by inhibition of their ROS detoxification capacity [30].

Impaired immune response and refractoriness to CIT in CLL is strongly influenced by neoplastic cells that do not rely on mitochondrial respiration (and energy supply). Such cells can survive and expand in a hypoxic microenvironment. When circulating in the blood under normoxic conditions, CLL cells display increased mitochondrial energy production [31]. However, once CLL cells are exposed to hypoxia they can switch to anaerobic metabolism [32]. The latter enables them to enter hypoxic niches in lymph nodes [33], bone marrow (BM) and the spleen (where oxygen levels range from 0%–4%) [34] and to re-cycle to the blood [32]. Eradication of minimal residual disease (MRD) in CLL is in particular difficult in the lymph nodes, where oxygen levels are much lower than in the blood or BM [35]. Taken together there is strong evidence that leukemia cells capable of growing in an hypoxic environment resist elimination by the immune system or CIT.

Here we provide a functional qualitative assay that can detect cells that are capable to grow well under hypoxia. It mimics the in vivo hypoxic niche by cultivating leukemic cells under low oxygen conditions and assessing the activity of glycolytic enzymes known for regulation of cellular growth under hypoxia. Enzymatic assays of PK and LDH activities are well established in laboratory diagnostics since decades. PK M2 is a typical marker of fast proliferating non-malignant and cancer cells. It redirects glucose carbons to nucleotide, protein and lipid synthesis reducing the doubling time of cancer cells under hypoxia [8,9]. Being the bottle neck for glucose flux, PK M2 and LDH regulate anaerobic anabolism and anaerobic energy supply [9]. Thus the activity of PK and LDH in the homogenates determines the presence of a neoplastic cell population with the potential to grow fast under severe hypoxia.

The test requires just five steps: blood collection, extraction of PBMCs by gradient centrifugation, cultivation of cells overnight, homogenization and assessment of enzymatic activity according to standard methods. Results are available overnight. Assessment of enzyme activities, in particular of PK and LDH used in our trial, is a robust, fast and well implemented technique in laboratories world-wide and thus this assay could help identifying high-risk CLL easily, thereby optimizing patient care. Our test provided both prognostic and predictive additional information, besides known risk factors in CLL. *IGHV*-U, del(11q) and del(17p)/*TP53* mutation correlate with shorter TFS [36]. Notably, the D-MS assay identified a subgroup of patients with favorable (prolonged TFS) clinical course within these risk groups. Moreover, patients harboring an *IGHV* mutation and having a HR D-MS had an unexpected, very aggressive clinical course. Consistently, in multivariate analysis, D-MS was found to be an independent factor of poor prognosis arguing that D-MS provides complementary prognostic information to genetic risk factors.

The C-statistics threshold level indicating clinical utility is c = 0.70 [37]. The C-statistics reached for the D-MS assay was c = 0.77 and thus higher than recently developed multiparameter risk models (c = 0.61–0.75) using biological markers [36], e.g. the CLL-IPI (International Prognostic Index) [38]. However, none of these parameters is predictive and can identify high-risk CLL. The only recommended predictive marker, del(17p)/*TP53* mutation, is highly specific (93%) but displays a poor sensitivity (13%), missing many high-risk CLL patients [36]. Here, the D-MS assay profoundly improves treatment decision making by identifying up to 5.5fold more high-risk CLL than the gold standard analysis for del(17p)/*TP53* mutation with a specificity of 75%–92%. As these patients may not benefit from CIT alternative treatment approaches with specific pathway inhibitors, e.g. BCRi, could be an option, as suggested by the investigation of BCRi response in our cohort. Results of this analysis with a heterogeneous cohort and limited patient numbers have to be interpreted with caution. However, these data suggest that D-MS HR CLL patients (representing clinically high-risk CLL) could benefit from BCRi front-line therapy.

Interestingly, new specific pathway inhibitors, e.g. Bcl-2 or BCRi, with excellent response rates in refractory CLL affect pathways promoting growth of cancer cells under severe hypoxia. Anoxia induces cell death by blocking the respiratory chain in the mitochondria to decrease the pro-survival signaling of Bcl-X_L_/Bcl-2. Consecutive Bax/Bak dependent release of cytochrome *c* results in Caspase-9 dependent apoptosis [39]. Thus, by inhibiting Bcl-2, venetoclax can specifically induce apoptotic cell death in hypoxic cells. Consistently other reports show that cytotoxic activity of Bcl-2-inhibitors ABT-737 and ABT-199 (venetoclax) in primary CLL cells is significantly increased in hypoxia (compared to normoxia), importantly, overcoming the resistance towards chemotherapy under hypoxia [40].

BCR signaling is required for leukemia cell proliferation. BTK and PI3K are down-stream components of the BCR pathway. Increased activity of intracellular tyrosine kinases leads to a switch to anaerobic glycolysis in cancer cells [41]. Allosteric binding of accumulated phosphotyrosine peptides or phosphorylation of Y105 of the allosteric center of PK M2 enzyme strengthens PK M2 dimer activity and may enable cancer cell growth under severe hypoxia. This suggests that tyrosine kinase inhibitors, e.g. the BTK inhibitor ibrutinib, might affect PK M2 activity and cellular survival under hypoxia by reducing the amount of phosphotyrosine proteins. One clinical feature of ibrutinib treatment in CLL is the onset of lymphocytosis due to the mobilization of leukemia cells from the lymph nodes into the blood. One might speculate that ibrutinib may impair growth of leukemia cells in the hypoxic niche in the lymph node and forces them to reenter oxygenated blood. These findings constitute a body of evidence that hypoxic CLL cells may be susceptible to specific inhibition of Bcl-2 or the BCR pathway.

In summary, this pivotal study proposes that determination of the hypoxic growth pattern of leukemia cells may guide different treatment approaches. Future clinical trials are warranted to validate the D-MS score as a unique as well as complementary prognostic and predictive factor to influence therapy decision in CLL.

## Appendix A Supplementary data

Supplementary table with blank corrected endpoint data. Image 1

Supplementary Information Image 2
