# Supplementary material for: Distinct Activities of Glycolytic Enzymes Identify Chronic Lymphocytic Leukemia Patients with a more Aggressive Course and Resistance to Chemo-Immunotherapy
Source: eBioMedicine. 2018 Jun 5;32:125–33. doi: 10.1016/j.ebiom.2018.05.030 (PMC6021262; doi:10.1016/j.ebiom.2018.05.030)
Supplement: Supplementary file 2 — Supplementary Information [file mmc2.pdf]

Distinct activities of glycolytic enzymes identify chronic lymphocytic leukemia patients with a more aggressive course and resistance to chemo-immunotherapy

Georg Gdynia, MD[1], Tadeusz Robak, MD[2], Jürgen Kopitz, PhD[1], Anette Heller, PhD[1], Svetlana Grekova[1], Katarina Duglova[1], Gloria Laukemper[1], Monika Heinzel-Gutenbrunner, PhD[3], Cornelius Gutenbrunner, PhD[4], Wilfried Roth, MD[5], Anthony D. Ho, MD[6], Peter Schirmacher, MD[1], Michael Schmitt, MD[6,7], Peter Dreger, MD[6,7], and Leopold Sellner, MD[6,7].

[1]Institute of Pathology, University Hospital Heidelberg, Heidelberg, Germany

[2]Medical University of Lodz, Copernicus Memorial Hospital, Lodz, Poland

[3]Department of Child and Adolescent Psychiatry, University Hospital Marburg, Marburg, Germany

[4] MH Statistical Consulting, Marburg, Germany

[5]Institute of Pathology, University Medical Center of the Johannes Gutenberg University Mainz, 55131 Mainz, Germany

[6]Department of Medicine V, University Hospital Heidelberg, Heidelberg, Germany

[7]National Center for Tumor Diseases (NCT), German Cancer Consortium (DKTK), Heidelberg, Germany

Supplementary Information

Supplementary methods

Quantitative reverse transcriptase polymerase chain reaction (qRT-PCR)

The primer pairs used are:

18S:
5′-TTCAGGAATGGGATGGTCTA-3′ (forward),
5′-GAGTTGTTGCACTGCACTTG -3′(reverse);

5′-GGCCTTTAGCTCTGTTCCTC -3′ (forward),
5′-AATGGTATGGGGAATGTGTG -3′ (reverse),

Human Aldolase A:
5′-CTGAAGATTGGGGAACACAC -3′(forward),
5′-CCTTCCAGGTAGATGTGGTG -3′(reverse);

5′-ACAGTGGTGTGTGGTGTCGT -3′ (forward),
5′-GCCGACTCCCCCTTAAATAG -3′ (reverse);

Human Aldolase B:
5′-CGGCCAAAGGACAGTATGTT -3′ (forward),
5′-TCGAATTTCCAGGATTGGAG -3′ (reverse);

5′-TCGTGGTGGGAATCAAGTTA -3′ (forward),
5′-CCATTCTGCTGACAGATGCT -3′ (reverse);

Human Aldolase C:
5′-ATGGAGAAACCACCACTCAA -3′ (forward),
5′-AGGTTCCACAATAGGCACAA -3′ (reverse);

5′-ACTCCATACCACAGCCCTTG -3′ (forward),
5′-GCAATTTCTTCTGCCCTCAG -3′ (reverse);

Human Enolase 3:
5′-GGGAACCCTGACCTCATACT -3′ (forward),
5′-CCATACTTGGCCTTGATGAC -3′ (reverse);

Human Glucose Phosphate Isomerase (GPI):
5′-ACCAAGCTCACACCATTCAT -3′ (forward),
5′-GTTGATGAGCCCATTGGTAG -3′ (reverse);

Human Hexokinase 2:
5′-TGCCAAGCGTCTACATAAGA -3′ (forward),
5′-GCTCCATTTCTACCTTCATCC -3′ (reverse);

Human LDH A:
5′-AAGATGGCGACTGTGAAGAG -3′ (forward),
5′-CCGATCCAGTTCCTATGA TG -3′ (reverse);

5′-GGTGTCCCTTTGAAGGATCT -3′ (forward),
5′-TGCAGTCACTTCTTTGTGGA -3′ (reverse);

Human LDH C:
5′-TCTGTACTGATTGCGCCAAG -3′ (forward),
5′-CCAATGCAACATCAACAAGG -3′ (reverse);

5′-GATCTTCAGCATGGCAGTCT -3′ (forward),
5′-ATGGACTATGGCAGGAATGA-3′ (reverse);

Human phosphofructokinase liver:
5′-GCTCCGACACTGCTGTAAAT -3′ (forward),
5′-ATCTTCTCCGTCATGTGC TC-3′ (reverse);

5′-ATCTCCCATGGACACACAGT -3′ (forward),
5′-GGCGTGAATACCATAGATGC -3′ (reverse);

Human phosphoglycerate mutase 2:
5′-CATCTGCTACACGTCAGTGC -3′ (forward),
5′-TGTAGTAGGGGTGCTTCTCG -3′ (reverse);

5′-GTTTCTGTGGCTGGTTCGAT -3′ (forward),
5′-ACTCCATCTTGGCATCCTTG -3′ (reverse);

Human phosphoglycerate kinase 1:
5′-CTGTGGGGGTATTTGAATGG -3′ (forward),
5′-CTTCCAGGAGCTCCAAACTG -3′ (reverse);

Human Pyruvate kinase:
5′-AGAAGATCAACGCCTCACTG -3′ (forward),
5′-AAAGGAAGCTGTCACCCTCT -3′ (reverse);

5′-ACCCAACAGCACCAATTGTA -3′ (forward),
5′-AGTCACCATGAAGGGGTAGC-3′ (reverse);

Human triosephosphate isomerase 1:
5′-TTCTCCTTGGCTGAGAGATG -3′ (forward),
5′-GGTGGTTCACATACACAGCA -3′ (reverse).

Validation of the metabolic test procedure

The test procedure has been developed according to the In Vitro Diagnostic Directive 98/79/EC and the MS CLL test procedure was tested to meet the applicable essential requirements on performance as outlined. On this account the following performance parameters were determined: intra-assay precision, critical difference, and inter-assay precision. Furthermore, the robustness of the MS against an increased amount of non-cancerous lymphocytes in the sample was examined and the diagnostic sensitivity and specificity were calculated.

Two different tests were performed to determine intra-assay precision. At first the MS of 20 replicates of one normoxic and one hypoxic CLL cell homogenate (containing 2 µg total protein) were analyzed. The coefficient of variation (CV) was calculated as the ratio of the standard deviation to the mean value (CV = SD/xmean). The critical difference (CD), the smallest difference between sequential laboratory results which is associated with a true deviation, was calculated by “1.96x√2xstandard deviation” (~3xSD) of the same data set. Intra-assay precision was determined in two different MS regions, LR and HR. A pool of cell homogenates from 30 CLL patients with the original MS of 2 (LR) was prepared. By adding LDH enzyme to the hypoxic cell homogenate (spiking), the score could be increased to 3.3 (HR region). Both sample pools were analyzed by ten repeated measurements.

To determine the inter-assay precision two technicians tested two samples at two different measurement time points (8 a.m., 4 p.m.) on five consecutive days and used two different microplate-reader-devices (VICTOR X 2030, Perkin Elmer and FLUOstar Omega, BMG Labtec, Ortenberg, Germany) in two different laboratories of the University Hospital of Heidelberg using the patient pools which were used for the intra-assay precision experiment.

To examine if the content of healthy lymphocytes influences the MS determination, the pool of 30 CLL sample homogenates was “contaminated” with a pool of PBMCs from 20 healthy donors. Buffy coats were obtained from the blood bank of the Institute for Clinical Transfusion Medicine and Cellular Therapy (IKTZ), Heidelberg, Germany. The amount of cancer cells in the tested CLL blood sample was close to 100%. Appropriate protein amounts of the sample pools were mixed in different ratios. In one case the mixtures were used in their original conditions (low risk region), while the MS was artificially increased by spiking the hypoxic cell homogenate with LDH enzyme (high risk region) in the other case.

Performance characteristics of the metabolic score

No change in activity results in a MS of 2.0 (see methods). Any significant change beyond 2.0 allows a qualitative assessment of whether there is a measurable subpopulation of cells that grows fast under hypoxia. Within-run precision was determined by 10-20 repeated measurements of un-pooled or pooled samples and showed coefficients of variation (CVs) of 6.2%-6.8% (Low=Low Risk range) and 5.9% (High=High Risk range) (**Supplementary Table 2**). Critical difference was derived from within-run variation and determined as 2.0±0.3 (2.0 (no change in activity) ± ~3xSD). Thus CLL with scores below 1.7 or above 2.3 were defined as MS HR, within 1.7 and 2.3 as MS LR. Day-to-day precision exhibited CVs of 4.3% (Low=LR range) and 9.9% (High=HR range), the higher inter-assay variation in the MS HR range, however, never resulted in a false classification (100% accuracy) and thus was considered acceptable (**Supplementary Table 2**). In summary intra- and inter-assay results were reproducible and independent from the score region or variations of test conditions. Albeit leukemia cell counts in the PBMC fraction of CLL patients were between 90%-100% we tested whether a hypothetical ‘contamination’ with healthy lymphocytes would affect the MS. For CLL patients classified as LR the MS did not differ from the MS of healthy blood donors (around 2.0, pooled samples, see **Supplementary Table 3**) showing that cells and matrix from `non-malignant´ PBMCs do not alter but resemble metabolism (regarding the MS) of CLL cells from LR patients. For HR CLL patients (n=30, pooled samples) even up to 40% ‘contamination’ of samples with healthy lymphocytes from 20 blood donors still resulted in a correct MS classification in HR or LR respectively (**Supplementary Table 4**).

Supplementary tables

Supplementary table 1. Pharmacological modification of enzymatic activities (OD decrease) and metabolic score (MS) in Mec-1 cells. 10^6 Mec-1 cells treated with 100µM PM2-tide and 25µM DASA (n=2, 24 h, both concentrations adjusted to high number of cells compared to cytotoxicity experiments performed with 10^4 cells in 96-well format). MS reference value 2.0±0.3.

Mec-1 sample	O2	PKla [OD]	PKha [OD]	LDH [OD]	MS	Fludarabine resistant [y/n]
**control**	Normoxia	0,61	0,13	0,38	1,95	
**control**	Hypoxia	0,65	0,20	0,72		No
**P-M2tide**	Hypoxia	0,84	0,11	0,89	4,58	Yes
**DASA**	Hypoxia	0,59	0,25	0,63	1,37	Yes

Supplementary table 2: Metabolic scores of reach individual patient. Patient with a metabolic score between 1.7 and 2.3 were classified as metabolic low risk (LR), patients with a metabolic score <1.7 or >2.3 were classified as metabolic high risk (HR).

Patient	Metabolic Score	Risk stratification	Patient	Metabolic Score	Risk stratification
P1	2,01	LR	P49	2,13	LR
P2	2,18	LR	P50	2,00	LR
P3	2,19	LR	P51	1,73	LR
P4	1,70	LR	P52	2,06	LR
P5	1,95	LR	P53	1,93	LR
P6	1,96	LR	P54	1,73	LR
P7	2,06	LR	P55	2,27	LR
P8	1,73	LR	P56	2,27	LR
P9	1,90	LR	P57	1,72	LR
P10	2,10	LR	P58	1,89	LR
P11	2,36	HR	P59	2,03	LR
P12	1,60	HR	P60	2,14	LR
P13	2,34	HR	P61	1,82	LR
P14	1,69	HR	P62	1,73	LR
P15	1,38	HR	P63	2,24	LR
P16	1,27	HR	P64	1,97	LR
P17	2,36	HR	P65	1,81	LR
P18	1,00	HR	P66	1,72	LR
P19	2,43	HR	P67	1,82	LR
P20	2,58	HR	P68	1,78	LR
P21	1,60	HR	P69	2,01	LR
P22	1,95	LR	P70	1,78	LR
P23	1,49	HR	P71	1,87	LR
P24	2,30	HR	P72	2,28	LR
P25	2,62	HR	P73	2,03	LR
P26	1,33	HR	P74	1,12	HR
P27	1,70	LR	P75	1,61	HR
P28	1,98	LR	P76	1,69	HR
P29	1,95	LR	P77	1,62	HR
P30	3,40	HR	P78	2,43	HR
P31	1,43	HR	P79	2,41	HR
P32	1,40	HR	P80	3,17	HR
P33	1,95	LR	P81	1,26	HR
P34	2,04	LR	P82	1,28	HR
P35	1,99	LR	P83	1,68	HR
P36	1,72	LR	P84	1,27	HR
P37	0,96	HR	P85	1,67	HR
P38	1,08	HR	P86	2,33	HR
P39	1,69	HR	P87	2,37	HR
P40	3,45	HR	P88	2,45	HR
P41	2,08	LR	P89	1,66	HR
P42	1,76	LR	P90	1,43	HR
P43	2,19	LR	P91	3,35	HR
P44	1,83	LR	P92	1,40	HR
P45	1,76	LR	P93	1,44	HR
P46	1,76	LR	P94	1,50	HR
P47	1,77	LR	P95	1,22	HR
P48	1,72	LR	P96	0,78	HR

Supplementary table 3: Metabolic Score provides best results for prediction of response to CIT. Correlation of absolute values and absolute value change of single enzymes (mU/mg) and metabolic score (MS) in homogenates of PBMCs from CIT treated (n=29) CLL patients (upon cultivation under hypoxic conditions) with refractory CLL.

Test Result Variable(s)	Area	Std. Error[a]	Asymptotic p-value[a]	Asymptotic 95% CI Lower Bound	Upper Bound
PK la hypoxia [mU/mg]	.664	.103	.138	.462	.866
PK la normoxia [mU/mg]	.547	.115	.674	.321	.772
PK ha hypoxia [mU/mg]	.598	.112	.376	.378	.818
PK ha normoxia [mU/mg]	.598	.108	.376	.387	.810
LDH hypoxia [mU/mg]	.652	.104	.170	.449	.855
LDH normoxia [mU/mg]	.667	.104	.132	.463	.870
PK la activity change after hypoxia	.738	.095	.032	.552	.924
PK ha activity change after hypoxia	.529	.110	.790	.315	.744
LDH activity change after hypoxia	.583	.110	.452	.368	.799
MS-Deviation	.770	.098	.015	.577	.963

a. Null hypothesis: true area = 0.5

Supplementary table 4. Intra-Assay (within-run) and Inter-Assay (day-to-day) precision.

MS Range	Mean	SD	CV [%]	Run	Patients
Low	1.85	0.12	6.2	20	1
Low	2.0	0.14	6.8	10	30
High	3.3	0.19	5.9	10	30

MS Range	Mean	SD	CV [%]	Days	Patients
Low	1.97	0.09	4.3	5	30
High	3.48	0.35	9.9	5	30

Abbreviations: SD = standard deviation; CV = coefficient of variation.

Supplementary table 5. Cell and matrix effects of PBMCs from healthy blood donors on classification of CLL Low Risk patients.

% of PBMCs from healthy blood donors (n=20)	% of PBMCs from CLL Low Risk patients (n=30)	mean	Correct classification LR vs HR
0	100	2.17	yes
10	90	2.30	yes
20	80	2.17	yes
30	70	2.08	yes
40	60	2.29	yes
50	50	2.05	yes
60	40	2.07	yes
70	30	2.06	yes
80	20	2.01	yes
90	10	2.06	yes
100	0	2.10	yes

Supplementary table 6. Cell and matrix effects of PBMCs from healthy blood donors on classification of CLL High Risk (HR) patients.

% of PBMCs from healthy blood donors (n=20)	% of PBMCs from CLL High Risk patients (n=30)	mean	Correct classification LR vs HR
0	100	5.05	yes
10	90	3.13	yes
20	80	3.12	yes
30	70	2.45	yes
40	60	2.67	yes
50	50	2.17	no
60	40	2.33	yes
70	30	2.42	yes
80	20	1.88	no
90	10	2.75	yes
100	0	2.09	no

Supplementary figures

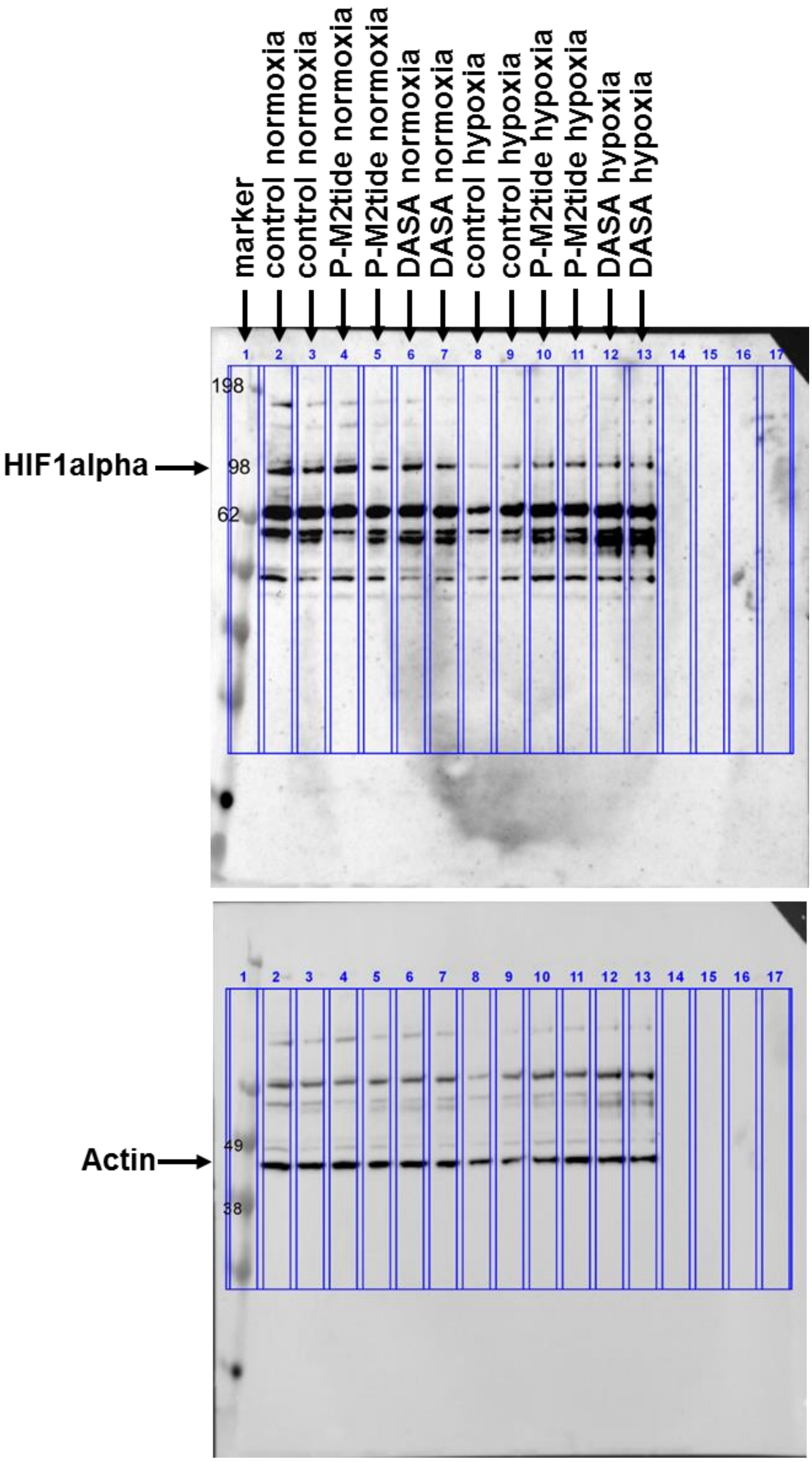

Supplementary figure S1. Uncropped western blot membranes from Mec-1 cells. Of note, equal amounts of protein were loaded for each normoxia and hypoxia sample pair to visualize potential differences in protein expression between the two conditions. HIF1-alpha was detected at ~100kDa.

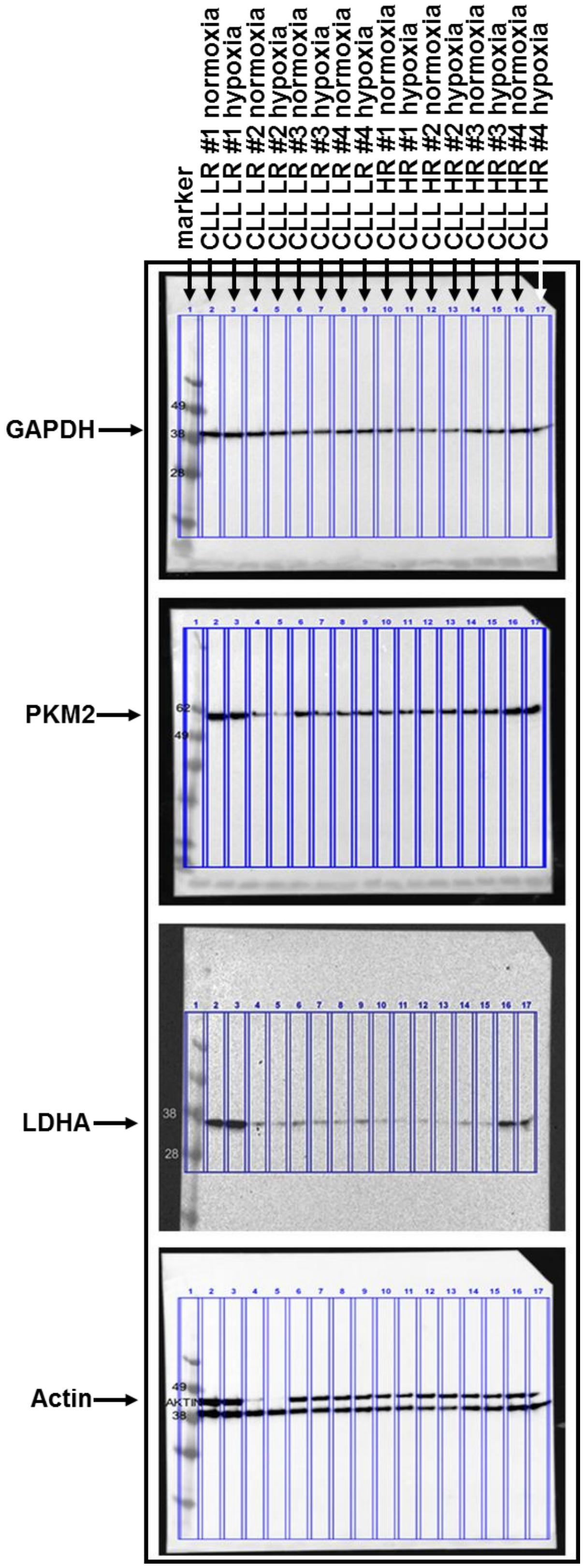

Supplementary figure S2. Uncropped western blot membranes from low and high risk CLL patients (according to Metabolic Score classification). Of note, equal amounts of protein were loaded for each normoxia and hypoxia sample pair to visualize potential differences in protein expression between the two conditions.

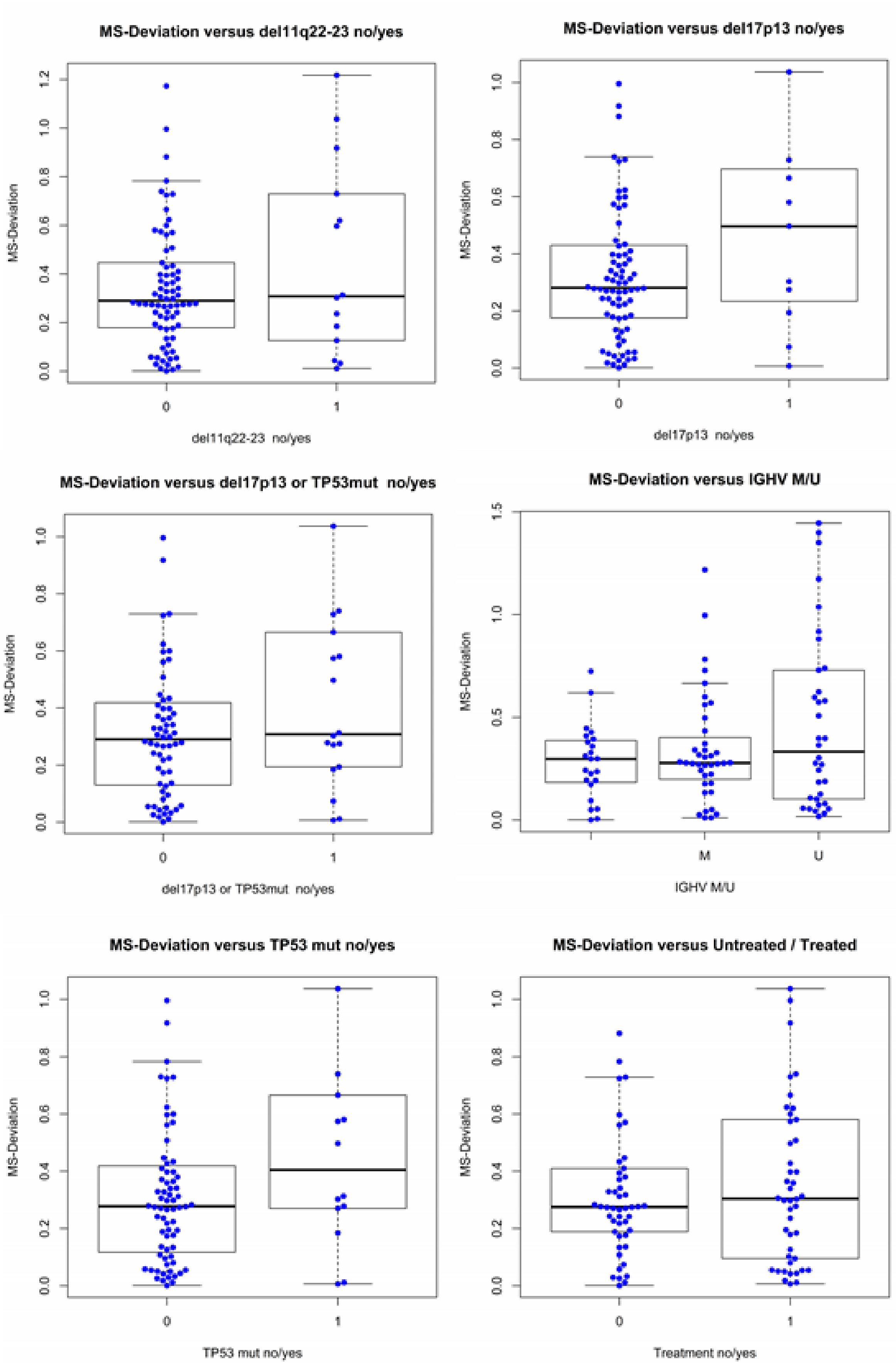

MS-Deviation versus del11q22-23 no/yes
MS-Deviation
0.0
0.2
0.4
0.6
0.8
1.0
1.2
0
1
del11q22-23 no/yes
MS-Deviation versus del17p13 no/yes
MS-Deviation
0.0
0.2
0.4
0.6
0.8
1.0
0
1
del17p13 no/yes
MS-Deviation versus del17p13 or TP53mut no/yes
MS-Deviation
0.0
0.2
0.4
0.6
0.8
1.0
0
1
del17p13 or TP53mut no/yes
MS-Deviation versus IGHV M/U
MS-Deviation
0.0
0.5
1.0
1.5
M
U
IGHV M/U
MS-Deviation versus TP53 mut no/yes
MS-Deviation
0.0
0.2
0.4
0.6
0.8
1.0
0
1
TP53 mut no/yes
MS-Deviation versus Untreated / Treated
MS-Deviation
0.0
0.2
0.4
0.6
0.8
1.0
0
1
Treatment no/yes

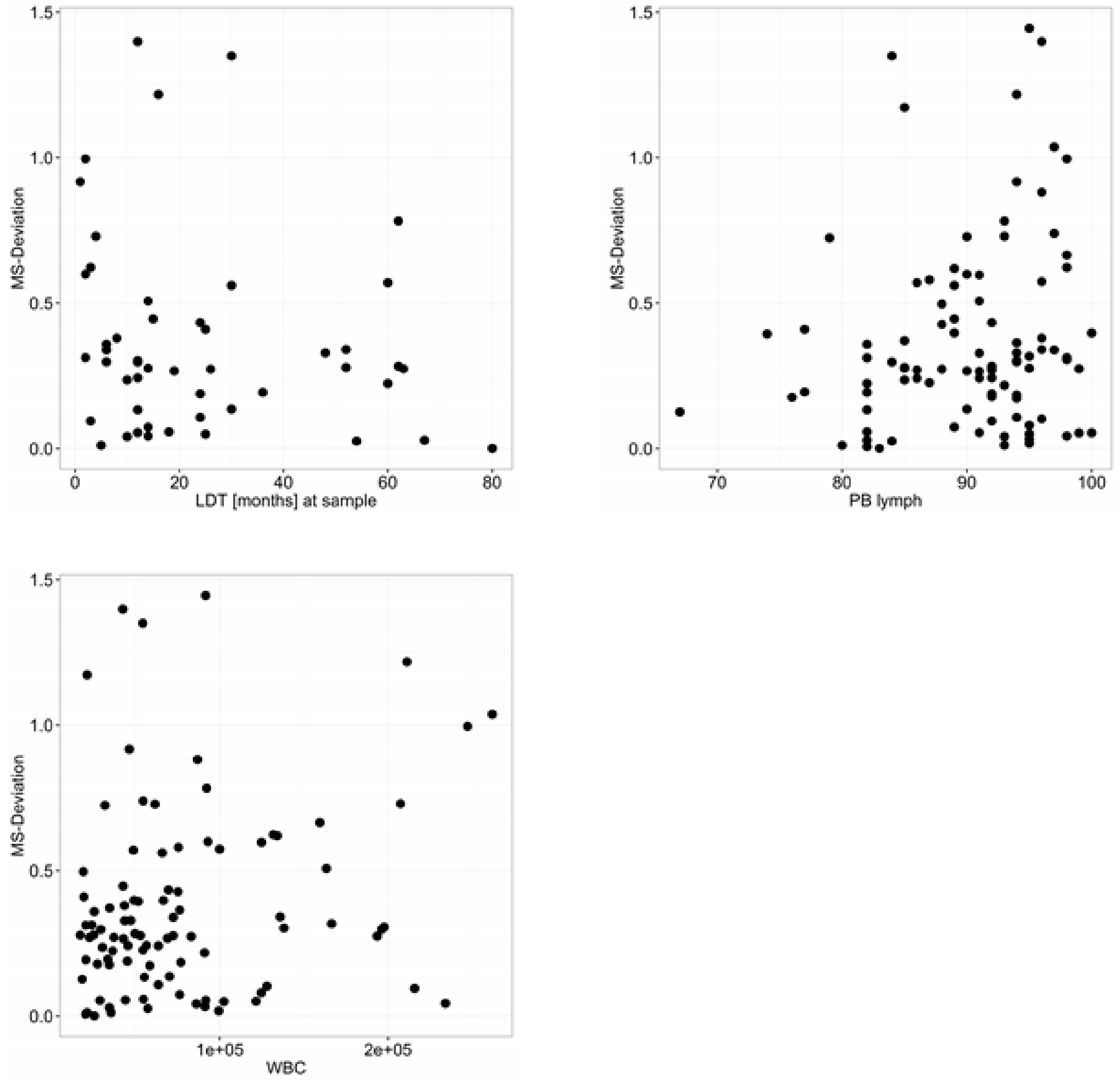

Supplementary figure S3. Correlation of clinical parameters with the metabolic score. Clinical parameters from the descriptive table of the main text showed no correlation with absolute metabolic score (MS) deviation.

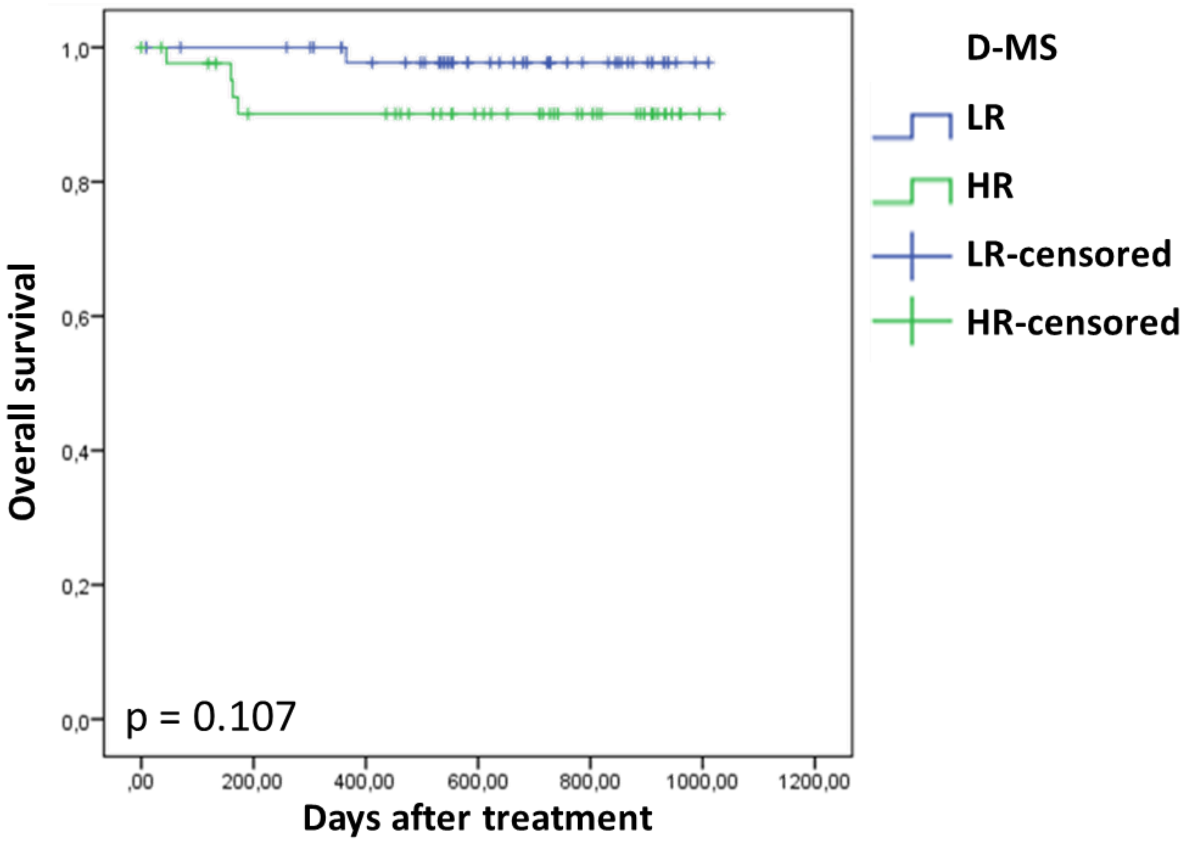

Supplementary figure S4. Overall survival. Comparison of overall survival of CLL patients classified as “high risk” (HR; n=45) and “low risk” (LR; n=51) according to the dichotomized metabolic score (D-MS). There were 5 deaths in the HR group and 1 death in the LR group. Due to the low number of events this difference was not significant (p=0.107).
